# Prosthetics

**Published:** 1894-02

**Authors:** J. M. Walker


ARTICLE V.

### PROSTHETICS.

REPORTED BY MRS. J. M. WALKER.

Read before the Alabama Dental Association.

Dr. J. Y. Crawford said there frequently seems to be a disposition to relegate mechanical dentistry to the background as if beneath our attention. But the best dentist is the all-round dentist; the one who is able and ready to do whatever is best for each patient. There is often great responsibility attached to the proper construction of an artificial denture. Of the two, a plate for the lower jaw ought to be the most comfortable. Yet how often is this the case? Have a good metal lining, or swaged aluminum with rubber attachments. Use a die and counter die of Babbitt's metal, in a sand mold, from a plaster impression, and you can swage even an aluminum plate that will fit like a wafer. Examine it in the mouth. Scallop it out when it wears, till, when the mouth is opened as in taking food, it will not rise up. Wax on the teeth and put it in the mouth as a base plate, and get the bite and occlusion. Adjust the teeth perfectly, flask it and vulcanize, putting in just enough rubber to fill the mold. The smooth aluminum will enable you to open and impact it, and get in just the right quantity of rubber not to drive in the plaster or disarrange the teeth. Cover all the upper aluminum surface with rubber, but let the aluminum rest on the tissues. This will give the greatest satisfaction, especially with patients who have tried to wear lower plates of rubber, celluloid, etc. For comfort I would use it in preference to continuous gum. For the upper jaw, make a good gold plate with rubber attachments.

For the benefit of the younger men, Dr. T. M. Allen gave in detail the method of swaging a gold plate, such as were made by the fathers, before rubber was heard of, saying that if we would more frequently advise gold plates we would educate the people up to the standard.

Dr. W. G. Browne described his greatly simplified method of making gold plates with rubber attachments, without dies. He uses No. 30 gage pure soft gold, burnishing it directly on the plaster model, and stiffening by soldering a platinum wire all around the pieces, and flowing solder over to make a smooth surface. In a lower parital plate, when the eight anterior teeth were in position in the mouth, he makes the portion of the plate extending across the anterior teeth of a double plate of gold, with bits of platinum between the two gold plates, soldering the two plates only at the points where he placed the aluminum pieces. The rubber covering the lingual surface of the piece entering between the two thicknesses of gold made a very strong piece without swaging or using dies in its construction.

Dr. Crawford said that the description of Dr. Browne's method was interesting, but he must protest against the idea of swaging gold over a plaster model with the fingers. If you want to make a gold plate, charge $100 or $150 for it and make it in conservative style, giving your time and labor to its honest construction. Swaging between metal dies is the only way to secure perfect accuracy.

Dr. Browne's method he considered only applicable to a collar or band for a tooth.

Dr. C. V. Rosser spoke of the great difficulty of swaging an upper plate when the arch is very high. He would swage it in two parts, the roof of the mouth, and the alveolar ridge, soldering together afterwards.

Dr. Chisholm said that to secure perfect accuracy it would be found necessary to have several sets of dies. In case of undercuts cut out a V and solder.

Dr. R. R. Freeman advocated the reliable old-fashioned die and counter die of zinc and lead. He said it is a well known fact that the expansion of plaster and the contraction of zinc mutually counteract each other, making an equipoise and giving a most accurate fit. Do not follow blindly after any one, but "think, and give a reason for the *think* that is within you!" For lower plates afford a bearing for the lips to press the plate down. Ridge or no ridge makes no difference in the lower jaw, unless it is of the hour-glass shape from great absorption at the roots of the teeth, leaving it full at the top. In such a case take the impression in modeling compound, and let it drag till it comes out. From an impression thus removed you can get a model on which to make a plate that will fit comfortably.

To reach an estimate of the comparative merits of the different die metals, Dr. Crawford proposed that at the next meeting of the Association Dr. Freeman and himself respectfully should make a metal plate. He would use Babbit's metal, Dr. Freeman using zinc and lead. They would each take an impression of the same mouth, and make two plates, each one in his own way. The plates to be tested on the cast and in the mouth for accuracy of fit.

Dr. Freeman expressed his willingness to accept this challenge, saying he felt assured in advance that while Dr. Crawford's might fit the cast, his would fit the mouth more perfectly.

Dr. Chisholm would use type metal as giving the most clean-cut, with lead for the counter die.

Dr. R. C. Young said that to get an accurate adjustment of a Logan crown he found it useful to take an impression of the exposed end of the root after it has been proximately trimmed, and make a cast. Smoke the cast, and on applying the crown you see exactly where it requires grinding.

Dr. Freeman paints the end of the root red. The pigment adhering to the crown shows where to touch it up with much less trouble than making a cast.

Dr. C. L. Boyd: When a patient wearing an upper plate has the anterior lower teeth in place, but will not wear a lower plate, the lower teeth often cut out the rubber, exposing the pins of the upper teeth. To prevent this fit small triangular pieces of gold or aluminum against the lingual surfaces of the upper front teeth. The lower teeth striking against the metal prevents exposure of the pins. Dr. Boyd uses a little rouge and glycerin mixed on his cement slab in adjusting artificial crowns.

Dr. Chisholm asked for ideas as to the best method of supplying an upper lateral incisor, all of the other teeth being in place and in good order.

Dr. Crawford suggested several methods—a cantilever bridge supported by cutting a hole in the cuspid, but no band around the front of the latter, or by a hood crown on the cuspid of gold coin swaged on a metal die, or of 22k. clasp metal, or the cuspid could be cut off, putting on a Richmond crown with an extension bridge lateral.

The officers of the Alabama Association are: Dr. T. M. Allen, Enfaula, President; Dr. R. A. Rush, Selma, First Vice-President; Dr. W. E. Proctor, Sheffield, Second Vice-President; Dr. S. W. Foster, Decatur, Secretary; Dr. G. M. Rousseau, Montgomery, Treasurer.—*Items of Interest.*

